# Evidence That the Blood Biomarker SNTF Predicts Brain Imaging Changes and Persistent Cognitive Dysfunction in Mild TBI Patients

**DOI:** 10.3389/fneur.2013.00190

**Published:** 2013-11-18

**Authors:** Robert Siman, Nicholas Giovannone, Gerri Hanten, Elisabeth A. Wilde, Stephen R. McCauley, Jill V. Hunter, Xiaoqi Li, Harvey S. Levin, Douglas H. Smith

**Affiliations:** ^1^Department of Neurosurgery, Center for Brain Injury and Repair, Perelman School of Medicine, University of Pennsylvania, Philadelphia, PA, USA; ^2^Department of Physical Medicine and Rehabilitation, Baylor College of Medicine, Houston, TX, USA; ^3^Department of Radiology, Baylor College of Medicine, Houston, TX, USA; ^4^Department of Neurology, Baylor College of Medicine, Houston, TX, USA; ^5^Michael E. DeBakey Veterans’ Affairs Medical Center, Houston, TX, USA; ^6^Department of Pediatrics, Baylor College of Medicine, Houston, TX, USA; ^7^Department of Pediatric Radiology, Texas Children’s Hospital, Houston, TX, USA

**Keywords:** surrogate marker, concussion, calpain, DTI, spectrin, diffuse axonal injury, prognostic marker, cognitive impairment

## Abstract

Although mild traumatic brain injury (mTBI), or concussion, is not typically associated with abnormalities on computed tomography (CT), it nevertheless causes persistent cognitive dysfunction for many patients. Consequently, new prognostic methods for mTBI are needed to identify at risk cases, especially at an early and potentially treatable stage. Here, we quantified plasma levels of the neurodegeneration biomarker calpain-cleaved αII-spectrin N-terminal fragment (SNTF) from 38 participants with CT-negative mTBI, orthopedic injury (OI), and normal uninjured controls (UCs) (age range 12–30 years), and compared them with findings from diffusion tensor imaging (DTI) and long-term cognitive assessment. SNTF levels were at least twice the lower limit of detection in 7 of 17 mTBI cases and in 3 of 13 OI cases, but in none of the UCs. An elevation in plasma SNTF corresponded with significant differences in fractional anisotropy and the apparent diffusion coefficient in the corpus callosum and uncinate fasciculus measured by DTI. Furthermore, increased plasma SNTF on the day of injury correlated significantly with cognitive impairment that persisted for at least 3 months, both across all study participants and also among the mTBI cases by themselves. The elevation in plasma SNTF in the subset of OI cases, accompanied by corresponding white matter and cognitive abnormalities, raises the possibility of identifying undiagnosed cases of mTBI. These data suggest that the blood level of SNTF on the day of a CT-negative mTBI may identify a subset of patients at risk of white matter damage and persistent disability. SNTF could have prognostic and diagnostic utilities in the assessment and treatment of mTBI.

## Introduction

Mild traumatic brain injury (mTBI), alternatively referred to as concussion, is the most common neurological injury and affects over 1.5 million children and adults each year in the United States alone, and hundreds of thousands of military personnel worldwide (1, 2). mTBI is typically undetectable with computed tomography (CT), yet can elicit long-term and clinically significant brain dysfunction in ∼15–30% of cases (3–6). Histopathological and biomechanical findings in experimental animal models and human cases that have come to autopsy suggest that the main underlying structural correlate for long-term functional impairment after mTBI is diffuse axonal injury (DAI) resulting from head rotational acceleration at the moment of injury (6–10). Developing neuroradiological methods, such as diffusion tensor imaging (DTI), have shown promise for the detection of white matter structural abnormalities after mTBI, but collectively these studies have yielded inconsistent results (11, 12). Consequently, new approaches are urgently needed for the rapid identification of mTBI patients who are at risk of suffering brain damage and persistent disability. In particular, a prognostic marker for mTBI with negative long-term functional consequences would enable the clinical evaluation of candidate neuroprotective treatments by identifying a set of patients most appropriate for trial enrollment.

Blood-based biomarkers for brain damage have long been evaluated as potential prognostic measures in mTBI, but none have emerged thus far as a means of identifying those cases of mTBI with evolving brain damage leading to long-term dysfunction at an early and potentially treatable stage. For example, a number of proteins expressed predominantly in the nervous system become detectable in the blood during the acute post-injury period in subsets of mTBI cases. Blood levels of the astrocyte-enriched proteins S100β and glial fibrillary acidic protein (GFAP), along with the neuron-enriched neuron-specific enolase (NSE), ubiquitin C-terminal hydrolase L1 (UCH-L1), and a proteolytic fragment of tau are reportedly elevated following injuries categorized as mild based on clinical examinations using the Glasgow Coma Scale (13–20). However, these studies either demonstrated the lack of a prognostic relationship between blood biomarker elevations and brain functional outcomes (13–18) or focused predominantly on TBI cases that also show head CT abnormalities (19, 20) and would be classified with moderate TBI or “complicated” mTBI at most centers. There is evidence that positive CT findings are themselves prognostic for poorer long-term outcome after TBI (21), and additional prognostic utility for blood markers for “complicated” mTBI remains to be established. For the much more common instances of CT-negative mTBI, blood-based markers for brain injury that are strong predictors of structural damage and long-term functional outcome have yet to be discovered (22, 23).

Given the challenge of early prognosis of functionally disruptive CT-negative mTBI, it is important to evaluate new blood-based biomarkers for neurodegeneration, and explore whether a combination of biomarkers, advanced neuroimaging, and neuropsychological methods may be more effective when used together than any single approach. Toward this end, we have discovered and begun to characterize several new candidate neurodegeneration biomarkers for acute brain damage, based on proteins that are released from degenerating neurons (24). Two of the released proteins are calpain-cleaved αII-spectrin proteolytic fragments [N-terminal fragment SNTF, originally referred to as BDP3 (25); and C-terminal fragment SBDP150, originally referred to as BDP1 (25, 26)]. These spectrin derivatives are mechanism-based markers for the calpain-associated necrotic mode of neurodegeneration especially prevalent in the brain after TBI or cerebral ischemia (26–29). αII-Spectrin N-terminal fragment (SNTF) increases in human blood after severe TBI (30) but has yet to be evaluated in mTBI. In this study, we examined the levels of SNTF in human plasma from CT-negative mTBI, orthopedic injury (OI), or uninjured control (UC) participants. By combining acute evaluation of this neurodegeneration biomarker with DTI and 3 months of neurobehavioral analyses, we examined whether SNTF might be a marker for mTBI and, if so, whether its blood levels relate to white matter abnormalities and persisting functional disability.

## Materials and Methods

### Study participants

The Institutional Review Boards of the University of Pennsylvania and Texas Medical Center, Houston, reviewed and approved the study. All participants in this study provided written informed consent (or assent if written consent was given by the minor’s parent) and were recruited and assessed with approval from and according to the ethical guidelines of the Institutional Review Boards of the recruiting institutions. All procedures were conducted in accord with the ethical standards of the Helsinki Declaration of 1975, as revised in 2000 (31).

This study on neurodegeneration biomarkers examined 38 participants with plasma collected within 24 h of injury. Of those, 17 sustained mTBI, 13 sustained an OI, and 8 were UCs. These participants were part of a larger study comprising right-handed participants of ages 12–30 years, who were recruited and tested on neuropsychological and brain imaging measures at baseline (within 96 h of injury), and at follow-up sessions at 1 month (neuropsychological measures only) and 3 months. Participant recruitment was from a random, unselected series of patients admitted to emergency centers in the Texas Medical Center, Houston, including Ben Taub General Hospital, Texas Children’s Hospital, and Memorial Herman Hospital, or, for the UC group, from the greater Houston metropolitan area. The smaller biomarker study subgroup was selected randomly from the overall mTBI study, and did not differ significantly from the larger study sample on age, socioeconomic status (SES), race, gender, GCS score, or Extracranial Injury Severity Score (ISS).

The 17 participants providing plasma samples that experienced mTBI, as defined by criteria from the Centers for Disease Control, had an injury to the head from blunt trauma, acceleration, or deceleration forces with one or more of the following conditions: (1) observed or self-reported confusion, disorientation, or impaired consciousness, dysfunction of memory at the time of the injury, loss of consciousness lasting <30 min; and, (2) symptoms such as headache, dizziness, fatigue, irritability, and poor concentration soon after the injury. Additional inclusion criteria were a Glasgow Coma Scale score of 13–15 upon examination at an emergency center, no abnormal findings on head CT, duration of loss of consciousness for no more than 30 min, post-traumatic amnesia for <24 h, and an Abbreviated Injury Score (AIS) ≤3 and an ISS of <12 modified to exclude the head region. Comparator participants were of two cohorts. For one, participants with OI were recruited <96 h post-injury provided they met the following criteria: right-handed, 12–30 years old, no loss of consciousness, no post-traumatic amnesia, no overt intracranial injury, AIS <3 for any region of the body and an ISS ≤12, and a normal brain CT (if done). A second UC cohort consisted of eight healthy participants who had not sustained any injury, but were similar to the two injury groups in age, gender, and level of education.

Exclusions included non-fluency in either English or Spanish, failure to provide adequate contact information for scheduling follow-up assessments, blood alcohol level (200 mg/dL, previous hospitalization for head injury, pregnancy when screened prior to brain imaging, pre-existing neurologic disorder associated with cerebral dysfunction and/or cognitive deficit (e.g., cerebral palsy, mental retardation, epilepsy) or diagnosed dyslexia, pre-existing severe psychiatric disorder (e.g., bipolar disorder, schizophrenia), and contraindications to undergoing MRI. The OI comparison group was included to control for risk factors (32, 33) that predispose to injury, including pre-existing behavioral problems, learning disabilities, and family variables, along with a general trauma context similar to those with mTBI. The uninjured group was included to examine effects not due to injury and to compare injured patients to the general young adult population.

### Neurobehavioral assessments

Participants were administered tests of cognition and assessed for symptoms related to post-concussive injury. For comparison with neurodegeneration biomarker findings, we analyzed data from three domains, speed of processing, executive memory, and cognitive flexibility, along with symptoms of concussion. The analyses were conducted by investigators blinded to the plasma biomarker data.

Rivermead Post-Concussion Symptoms Questionnaire [RPCS; (34)]. The RPCS is a 16-item self-report of cognitive, emotional, and somatic complaints that are commonly reported following mTBI. Factor analyses have elicited a three-factor solution comprising cognitive, somatic, and emotional problems (35), although different factor structures have been reported (36). The participants were asked to rate the severity of each symptom (currently compared to pre-injury levels) from 0 – “not experienced at all” to 4 – “severe problem.” The primary variable was the total score.

Symbol-Digit Modalities Test [SDMT; (37)]. This is a timed substitution task with written and oral response modalities and is highly sensitive to processing speed deficits in the 8–78 year age range. Using a reference key, each examinee was asked to pair specific numbers with given geometric symbols within 90 s. The number of correct responses in the written modality was the variable used in this study.

KeepTrack task [KT; (38, 39)]. This updating task requires adding and deleting items in working memory according to semantic category, and the maintenance of semantic categorical representations. It has been validated in the mild TBI population (40). The variable used was the mean percent correct items per list recalled.

### Diffusion tensor imaging

All participants underwent MRI without sedation on a Philips 3.0 T Achieva scanner. Rigorous quality assurance testing was performed including American College of Radiology phantom testing: no concerns with quality assurance were noted during the course of the study.

An axial single-shot spin-echo echo-planar imaging sequence with 30 diffusion-encoding directions was used for DTI acquisition. Other parameters included a 256 mm field of view, an acquisition voxel size of 2 mm × 2 mm × 2 mm, repetition time of 11,526 ms, echo time of 51 ms, sensitivity encoding (SENSE) reduction factor of 2, two B factors (0 s/mm^2^ low B and 1000 s/mm^2^ high B), with two acquisitions to average the signal of the two DTI scans in order to ensure better signal-to-noise ratio. DTI acquisition consisted of 70 slices. A SENSE eight-channel head coil was used.

### Image processing

The corpus callosum, right and left uncinate fasciculi, and right and left frontal lobes were selected as structures of interest due to their known vulnerability in DTI studies of TBI and their presumed relation to the measures of speed of cognitive processing, memory updating, and executive function, and post-concussion symptoms. Additionally, DTI measurement of these structures has been shown to be reproducible both between and within raters on quantitative tractography using previously published protocols. In this study, DTI data were analyzed twice by a single rater to establish intra-rater reliability using intra-class correlational coefficients (ICCs). A subset of the images was analyzed by two raters to establish inter-rater reliability. ICCs for all measurements were above 0.95.

### Quantifying the neurodegeneration biomarker SNTF

The sandwich immunoassay for quantifying calpain-cleaved SNTF from human plasma is a modification of a method published previously (30, 41) in which the enzymatic amplification and detection steps of ELISA were replaced with next-generation electrochemiluminescence detection chemistry. Briefly, 96 well plastic microplates with an underside electrode (Meso Scale Diagnostics) were coated overnight with the capture antibody, a monoclonal directed at the SH3 domain in the N-terminal portion of the αII-spectrin subunit (D8/B7 @ 1/1000; Abcam). For the antigen capture step, human plasma samples diluted to 40% or SNTF standards (25 μls/well) prepared in 0.25% bovine serum albumin in Tris-buffered saline (pH 7.4) were added in triplicate for 2 h at 22°C. The detection antibody was a purified rabbit IgG prepared in our laboratory that is reactive with the calpain-generated neoepitope at the carboxyl-end of the stable calpain-derived α-spectrin ∼150 kDa N-terminal fragment (SNTF; 1/5,000). The specificity of this cleavage site-specific antibody for SNTF generated by calpain proteolysis has been well established by Western blot, protease digest, and protease inhibitor studies (24, 27, 30, 42, 43). The reporter probe was goat anti-rabbit IgG conjugated to ruthenium (SulfoTag, Meso Scale Diagnostics, Rockville, MD, USA; 1/500). In the presence of read buffer containing tripropylamine and application of current to the plate electrode, a chemiluminescent product is produced in proportion to the bound antigen. Chemiluminescent signals were quantified by a SECTOR Imager 2400 system (Meso Scale Diagnostics). Standard curves were generated using serial dilution of a preparation of α-spectrin partially purified from brain and digested with purified calpain I, essentially as described before (44). Briefly, the digestion was performed for 10 min at 30°C at a 300:1 ratio by weight of spectrin extract:calpain I in a buffer of 5 mM Tris-HCl (pH 7.8), 0.6 M KCl, 5 mM β-mercaptoethanol, 2 mM CaCl_2_. Purified bovine erythrocyte calpain I for the digest was obtained from Sigma (St. Louis, MO, USA). Reactions were quenched and the calpain I inactivated by addition of 5 mM EDTA followed by freeze-thaw. One unit of SNTF is defined as the signal derived from the SNTF standard diluted to 1 nl/ml, corresponding to ∼500 pg of the spectrin-containing brain extract starting material per ml.

Control experiments were performed to distinguish SNTF-related signals from non-specific signals emanating from heterophilic substances that are present in a subset of human plasma samples and confound attempts to measure very small amounts of target antigen (45). These control immunoassays were conducted as above, except that the detecting IgG specific for SNTF was replaced with normal IgG purified from pre-immune serum from the same rabbit. SNTF-specific signals were calculated as the difference between the specific and pre-immune detecting IgG signals and converted to standardized units. The minimum detection sensitivity was 10 units. The immunoassays were conducted and analyzed and the results replicated by investigators blinded to all other patient data.

## Results

### A subset of mTBI cases exhibit long-term impairment in cognitive function

A total of 38 randomly selected participants in an ongoing larger study of mTBI provided plasma samples on the day of injury for quantification of the neurodegeneration biomarker SNTF: 17 were diagnosed with mTBI and 13 with OI, whereas 8 were UCs. The biomarker study subgroup did not differ from the overall study group in terms of initial injury severity, age, gender, or other factors (Table 1). Among these 38 cases, brain structural integrity was assessed by DTI within 4 days of injury for 28 of the participants. Brain performance was evaluated by neuropsychological testing within 4 days of injury and at 1 and 3 months thereafter for 27–29 of the participants, depending on the test battery. The three biomarker study cohorts did not differ significantly from one another in age, gender, or level of education.

**Table 1:** **Representativeness of biomarker study subgroup relative to participants in the ongoing mTBI study**.

	Overall group mean (±SD) (*n* = 205)	Biomarker group mean (±SD) (*n* = 38)	*p*-Value
Age at baseline	20.2 (±5.4)	20.5 (±5.8)	0.80
SES	−0.0028 (±0.79)	−0.039 (±0.72)	0.80
Race % non-black	61	60	0.87
Gender % female	33	26	0.38
GCS (mTBI)% <15	23	24	0.85
Non-cranial injury severity	0.93 (±1.17)	1.37 (±1.42)	0.13

*There were no differences related to demographics or injury between the biomarker study group and the overall study group (*t*-test)*.

In comparison with the UC group, the mTBI group demonstrated overall cognitive performance deficits at 3 months post-injury on the SDMT, KT test, and the cognitive component of the RPCS, similar to reports from other studies [e.g., Ref. (15, 40, 46)]. Notably, performance deficits were also observed in the OI group relative to the UCs (40). Neuropsychological test performance varied widely among the mTBI participants: some performed indistinguishably from the UC group at both early and late time points, while other participants showed impairments at the acute and/or 1 month time point that resolved by 3 months, and a third set exhibited dysfunction persisting out to 3 months.

### Plasma SNTF is elevated in a subset of mTBI cases

We evaluated SNTF as a candidate plasma biomarker for human mTBI. This αII-spectrin fragment is generated by the calpain family of cysteine proteases (25, 27, 44) and accumulates in axons damaged by stretch injury *in vitro* or TBI *in vivo* (29, 47–50). It is released from neurons upon plasma membrane disruption (24). Here, plasma SNTF measured on the day of injury was at least twice the lower limit of detection of 10 units in an ultrasensitive sandwich immunoassay in a subset of participants, 7 of 17 mTBI cases and 3 of 13 OI cases. In contrast, plasma SNTF was below 10 units in all 8 healthy UC participants. The immunoassay signals from the positive plasma samples were confirmed as being specific for SNTF, and not from heterophilic substances that confound human plasma biomarker studies (45), by control experiments in which the SNTF-specific detecting IgG was replaced with pre-immune IgG isolated from the same rabbit. The SNTF positive mTBI participants suffered injuries spanning a variety of mechanisms from sports, assault, motor vehicle/motorcycle crashes, falls, and being struck by a falling object. Among the SNTF positive participants, the plasma sampling time varied from 1 to 24 h post-injury (median 14 h), and the absolute SNTF level ranged from 20 to 150 units. The SNTF positive and negative groups did not differ significantly from one another in age or gender.

### Elevated plasma SNTF on the day of injury is related to white matter damage and long-term cognitive dysfunction

To examine the relationship between plasma SNTF levels on the day of mTBI and DAI, the 28 participants among the mTBI, OI, and UC cohorts with usable neuroradiological data were dichotomized as either SNTF positive or negative, and the two groups were evaluated comparatively for white matter structural abnormalities by DTI. Compared with the 19 SNTF negative cases analyzed by DTI within 4 days of injury, the 9 SNTF positive cases (7 mTBI and 2 OI) as a group exhibited significant reductions in FA and increases in ADC in the corpus callosum and uncinate fasciculus (Table 2). The FA and ADC are thought to quantify the orientation and structural integrity of white matter axons, and their differences as a function of dichotomized plasma SNTF levels provide evidence that plasma elevations in this neurodegeneration biomarker after injury may be related to DAI.

**Table 2:** **Plasma SNTF is related to diffusion tensor imaging differences in select white matter tracts**.

Region/metric	Mean (SD) all SNTF−(*n* = 19)	Mean (SD) all SNTF+ (*n* = 9)	*p*-Value	Effect size
Corpus callosum				
FA	0.496 (0.02)	0.479 (0.01)	0.034	0.91
ADC	0.821 (0.03)	0.839 (0.02)	0.13	0.63
Uncinate fasciculus, left				
FA	0.405 (0.02)	0.388 (0.02)	0.09	0.73
ADC	0.754 (0.03)	0.775 (0.03)	0.14	0.63
Uncinate fasciculus, right				
FA	0.389 (0.01)	0.367 (0.02)	0.001	1.48
ADC	0.774 (0.02)	0.798 (0.03)	0.035	0.89
Frontal lobes, left				
FA	0.394 (0.02)	0.383 (0.02)	0.26	0.47
ADC	0.765 (0.02)	0.782 (0.02)	0.07	0.77
Frontal lobes, right				
FA	0.382 (0.03)	0.381 (0.02)	0.95	0.03
ADC	0.783 (0.02)	0.794 (0.02)	0.15	0.59

*Dichotomized plasma SNTF levels on the day of injury discriminate groups on brain white matter structural integrity indexed by diffusion tensor imaging performed within 96 h. Effect size is reported as Cohen’s *d*, where 0.2–0.49 reflects small, 0.5–0.79 medium, and 0.8 or higher large effect size, and *p*-value is from two-tailed *t*-test*.

Long-term behavioral studies have provided evidence that ∼15–30% of CT-negative patients with mTBI develop brain functional disability that can persist for many months post-injury (3–6). To examine the prognostic relationship between plasma SNTF levels measured on the day of mTBI and long-term brain function, participants were evaluated within 4 days and again at 1 and 3 months post-injury on a set of tests for cognitive performance and assessed for post-concussion symptoms. These included written and oral versions of the SDMT, which measures speed of cognitive processing and is a sensitive index of cognitive functioning independent of intelligence level (37), the KT (38, 39), a measure of memory updating and executive function, and the RPCS (34, 51), a self-report assessment of the severity of somatic, emotional and cognitive symptoms after concussion. For groups dichotomized with respect to plasma SNTF levels on the day of injury, there were marked differences in functional measures at both the acute and long-term time points. Plasma SNTF did not discriminate symptomatology on the overall RPCS, but showed an association with impairments in the cognitive and somatic components at 3 months post-injury that did not reach statistical significance. Most importantly, a level of plasma SNTF on the day of injury of >10 units discriminated test performance at 3 months on the SDMT and the KT, and the relationship with the former cognitive deficit was significant (*p* = 0.039; Table 3). The significant discrimination in the written and oral versions of the SDMT observed across all study participants based on dichotomized plasma SNTF (Table 3) was even stronger among the mTBI cases by themselves (written SDMT: SNTF+ = 46.8; SNTF− = 59.1; *p* = 0.011; oral SDMT: SNTF+ = 70.1; SNTF− = 53.3; *p* = 0.024).

**Table 3:** **Plasma SNTF on the day of a mTBI relates to impaired cognitive performance at 3 months post-injury**.

Test	All SNTF+	All SNTF−	Effect size
Symbol-digit modalities test, written (total correct responses)	52.00 (12.1)	63.47 (14.9)	0.88
KeepTrack task (percent correct recalled)	88.89 (7.8)	92.72 (5.6)	0.63
RiverMead post-concussion symptoms (total score)	9.44 (10.89)	6.37 (11.08)	0.28

*Dichotomized plasma SNTF levels on the day of injury (±SD in parentheses) are related to behavioral differences 3 months post-injury. Effect size is reported in Cohen’s *d*, where 0.2–0.49 reflects a small, 0.5–0.79 a medium, and 0.8 or higher a large effect size. The difference in cognitive performance on the written Symbol-Digit Modalities Test across all study participants based on plasma SNTF is significant by two-tailed *t*-test (*p* < 0.04), as is the difference within the mTBI cases by themselves (SNTF+ = 46.8; SNTF− = 59.1; *p* < 0.025)*.

**Figure 1:**
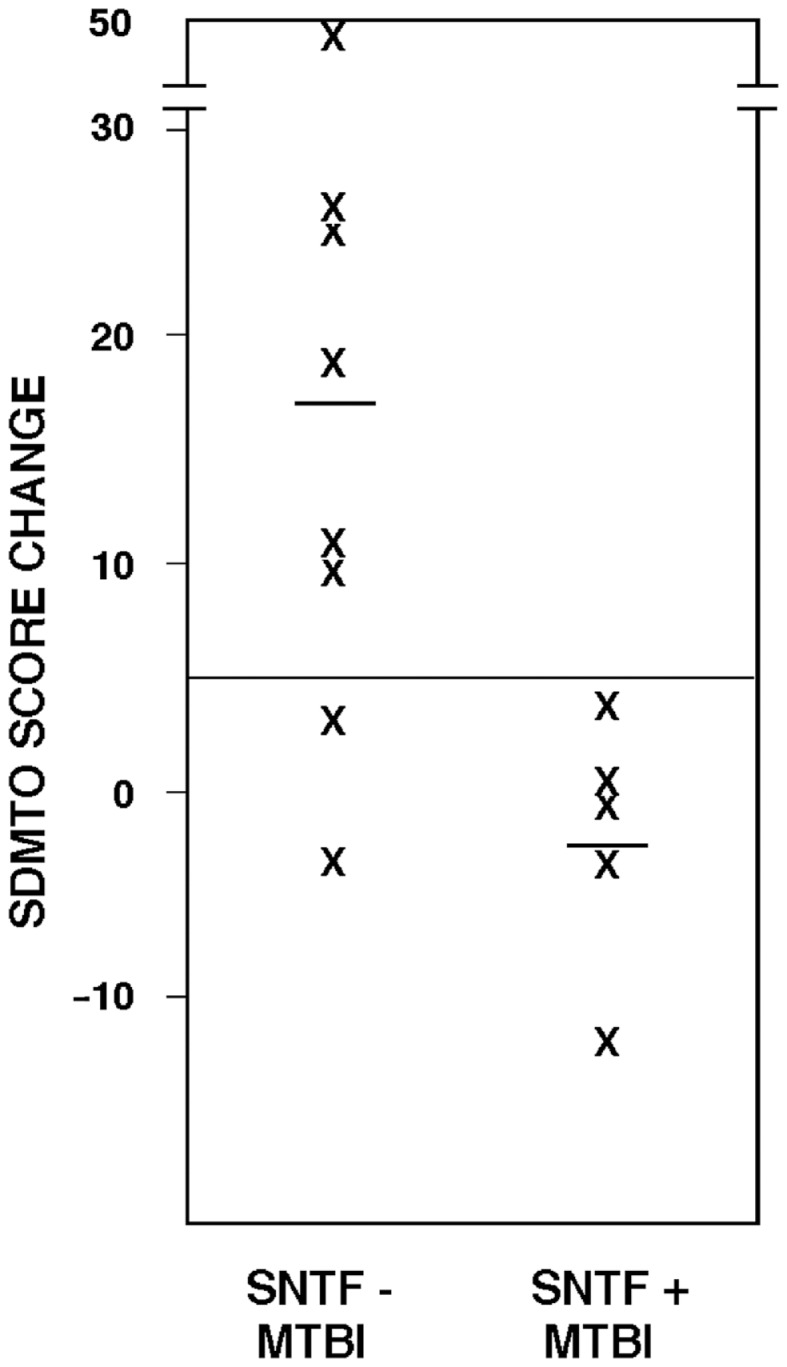
**Plasma SNTF discriminates 3 months changes in cognitive performance after mTBI**. The difference in SDMTO scores between the acute (1–4 day) and chronic (3 months) post-injury periods is plotted as a function of dichotomized plasma SNTF levels on the day of mTBI. The difference in cognitive performance recoveries between the biomarker negative and positive mTBI groups is significant (*p* < 0.03, two-tailed*t*-test).

Plasma SNTF on the day of mTBI also correlated with recovery of cognitive performance. Among the 13 mTBI participants evaluated by the oral SDMT in both the acute (1–4 days) and long-term (3 months) post-injury time periods, test scores for the SNTF – cases improved by 17 points (±5.7 SEM), whereas those for the SNTF+ cases worsened by 2.6 points (±2.7). The difference in 3 months recovery of cognitive performance as a function of dichotomized plasma SNTF levels was significant (*p* < 0.03). Six of eight SNTF− cases of mTBI showed improvement in cognitive performance over 3 months of five points or greater on the oral SDMT, compared with none of the five SNTF+ cases (Figure 1). Based on this preliminary *post hoc* assessment, plasma SNTF on the day of mTBI showed 100% sensitivity and 75% specificity for predicting failure to improve cognitive performance over the first 3 months after a CT-negative mTBI.

## Discussion

In this study, we provide evidence that the blood level of the neurodegeneration biomarker SNTF identifies mTBI patients on the day of their injury likely to have both white matter changes with advanced neuroimaging suggestive of DAI, and also cognitive dysfunction that persists for at least 3 months. Whereas a number of brain-enriched proteins have been evaluated before as candidate prognostic markers for cases of mTBI with negligible CT findings, including the astrocyte-enriched S100β and GFAP along with the neuron-enriched NSE, cleaved tau, a C-terminal fragment of αII-spectrin termed SBDP145, and UCH-L1, none has demonstrated a prognostic relationship with structural signs for white matter injury or functional signs for impaired cognition (17, 22, 23, 52). A recent study of candidate blood biomarkers in cases of repetitive low-level blast did not perform head CT evaluations, and demonstrated relationships with cognitive performance only after analyses were conducted *post hoc* on a subset of 10 subjects (53). In contrast to the prior findings with other marker candidates, our results raise the possibility that the blood level of SNTF and potentially other neurodegeneration biomarkers sampled in the acute period after CT-negative mTBI might help identify at an early and potentially treatable stage a subset of cases at risk of developing white matter tract structural damage and long-term disability.

αII-Spectrin N-terminal fragment is an especially plausible biomarker for DAI thought to underlie long-term brain functional impairments after mTBI (6–9, 54). It is generated by the calpain family of calcium-activated proteases (25, 27, 44), and is an N-terminal 1176 residue fragment of the α-subunit of brain spectrin (55), an actin-binding cytoskeletal protein that is abundant in axons (56, 57). SNTF is an established histological and biochemical marker for the necrotic mode of neurodegeneration driven by intraneuronal calcium overload and characterized by sustained calpain activation and the degradation of a host of functionally important cytoskeletal, cytoplasmic, and membraneous calpain substrates (58–60). This calpain-derived spectrin fragment accumulates in damaged axons after stretch-induced injury of cultured neurons (50) or after experimental TBI in animal models (29, 47, 49) as well as in the corpus callosum of human TBI patients (48). SNTF is released from dying neurons in response to a variety of neurodegenerative stimuli (24). Therefore, the injury-induced elevation in plasma SNTF in a subset of mTBI cases reported here is consistent with the hypothesis that functionally impactful mTBI triggers calpain activation and spectrin degradation within vulnerable axons, followed by efflux of the stable fragment SNTF into the brain parenchyma and bloodstream in association with the axon tract damage underlying brain functional impairment.

The relationship between dichotomized plasma SNTF on the day of injury and structural differences in the corpus callosum and uncinate fasciculus in the acute post-injury period (Table 2) supports this hypothesis. The SNTF positive group exhibits significant decreases in FA and increases in the ADC in the two white matter tracts, similar to the directional changes in measures of white matter integrity reported in the acute post-injury period in some but not all DTI studies of mTBI (6, 11, 12). These tracts are known to be susceptible to developing histopathology after TBI (8, 54). As the SNTF positive and negative groups do not differ significantly in age, the DTI differences as a function of this blood neurodegeneration biomarker are not simply the result of aging-dependent changes in white matter (61). The significant relationship between plasma SNTF and alterations in FA and ADC in the right uncinate fasciculus detectable with DTI is noteworthy, in light of the hypothesized key functional role for this white matter pathway connecting the limbic system to prefrontal regions (62), and the reported hemispheric functional differences in the tract (63).

Increased plasma SNTF post-concussion is related not only to structural evidence for DAI, but also functional evidence for long-term cognitive impairment. Whereas a subset of the participants with mTBI exhibit no discernible deficits on a battery of cognitive, somatic, or emotional tests post-injury, a second group shows performance deficits that resolve over time, while a third group develops impaired brain performance persisting for at least 3 months post-injury. Strikingly, the dichotomized plasma level of SNTF measured on the day of injury is related to cognitive dysfunction at 3 months, as evidenced by a significant deficit in the SNTF positive group in the SDMT and trends toward impairments in the KT test (Table 3) and the cognitive component of the RPCS (data not shown). The ability of plasma SNTF elevations to significantly differentiate long-term cognitive decline holds across all 28 participants in the mTBI, OI, and UC groups and even more strongly among the mTBI cases by themselves. Plasma SNTF on the day of mTBI also discriminated subsequent change in cognitive performance on the SDMT, with a positive SNTF finding predicting failure to improve cognitive performance over 3 months post-injury (Figure 1). In contrast to these long-term measures of cognitive function, plasma SNTF bears only a small relationship to the 3 months RPCS, a global assessment of cognitive, somatic, and emotional post-concussion symptoms. Further research will be required to define the precise relationships between plasma elevations of SNTF after mTBI and different dimensions of long-term brain performance.

The current study relating plasma SNTF to both structural and functional outcomes after mTBI is preliminary in nature and has several limitations, including a small sample size. For example, this study is insufficiently powered to address whether the absolute level of plasma SNTF might correlate with the severity of long-lasting behavioral dysfunction among participants functionally impacted by mTBI. The data reported here relating plasma SNTF to DTI changes in multiple axon tracts, coupled with evidence that this marker accumulates in damaged axons (47–50), strongly suggest but do not definitively establish that the plasma increase in SNTF after mTBI emanates from a central axonal source. The optimal plasma SNTF cutoff level for the prognosis of mTBI cannot be determined from these data alone, nor can the optimal time for measuring it post-injury or the precise sensitivity and specificity of the marker for functionally impactful mTBI. Plasma SNTF is also detectable in a small fraction (<25%) of the OI cases. In this regard, it is noteworthy that a recent study of orthopedic injuries by DTI and neurocognitive assessments raises the possibility that they sometimes lead to brain structural and functional abnormalities consistent with an undiagnosed mTBI (40). Whether the plasma rise in SNTF identifies an undiagnosed mTBI in cases of OI will require further study. Similarly, confirmatory prognostic studies in larger, independent cohorts of mTBI cases will be needed to determine definitively whether blood elevation in SNTF, either alone or perhaps in combination with other neurodegeneration biomarkers, serves as a reliable predictor of long-term outcome after CT-negative mTBI. Conceivably, there may be prognostic value in coupling plasma neurodegeneration biomarker findings with advanced neuroradiological and neuropsychological approaches for identifying mTBI patients at risk of developing brain damage and lasting disability, but this has yet to be established.

These unresolved issues take on increased urgency given the vital importance of early prognosis of functionally impactful mTBI. Despite being commonly associated with negligible head CT findings, mTBI is prevalent among both civilian and military populations and can lead to long-term brain dysfunction in as many as 15–30% of cases. A validated prognostic method for mTBI would have major utilities, including: (i) for stratifying mTBI cases and selecting a high risk population best suited for clinical trials of experimental neuroprotective treatment interventions; (ii) for serving as a surrogate endpoint for clinical neuroprotectant treatment trials; (iii) for rapidly identifying mTBI cases most likely to benefit from early initiation of rehabilitation therapies designed to improve functional outcomes; and (iv) for identifying sports and military participants at increased risk of further brain damage and disability. The current study relating SNTF to both radiological evidence for DAI and psychological evidence for long-term cognitive dysfunction raises the possibility that plasma neurodegeneration biomarkers such as SNTF may have important applications for the clinical evaluation and medical treatment of mTBI.

## Author Contributions

The research was conceived by Robert Siman, Harvey S. Levin, and Douglas H. Smith. Robert Siman and Nicholas Giovannone measured plasma levels of SNTF. Gerri Hanten conducted behavioral analyses of the study participants. Elisabeth A. Wilde, Stephen R. McCauley, and Jill V. Hunter conducted radiological analyses of the participants. Xiaoqi Li performed statistical analyses of the behavioral, radiological, and plasma biomarker data. Robert Siman, Gerri Hanten, Elisabeth A. Wilde, Stephen R. McCauley, Harvey S. Levin, and Douglas H. Smith wrote the manuscript.

## Conflict of Interest Statement

A provisional patent application has been filed by the University of Pennsylvania on the use of SNTF as a prognostic biomarker for concussion, with Robert Siman named as inventor. There are no other commercial or financial relationships that could be construed as a potential conflict of interest.
